# The RNA Methyltransferase METTL3 Promotes Endothelial Progenitor Cell Angiogenesis in Mandibular Distraction Osteogenesis via the PI3K/AKT Pathway

**DOI:** 10.3389/fcell.2021.720925

**Published:** 2021-11-01

**Authors:** Weidong Jiang, Peiqi Zhu, Fangfang Huang, Zhenchen Zhao, Tao Zhang, Xiaoning An, Fengchun Liao, Lina Guo, Yan Liu, Nuo Zhou, Xuanping Huang

**Affiliations:** ^1^Department of Oral and Maxillofacial Surgery, Hospital of Stomatology, Guangxi Medical University, Nanning, China; ^2^Guangxi Key Laboratory of Oral and Maxillofacial Rehabilitation and Reconstruction, Nanning, China; ^3^Guangxi Clinical Research Center for Craniofacial Deformity, Nanning, China; ^4^Guangxi Key Laboratory of Oral and Maxillofacial Surgery Disease Treatment, Nanning, China

**Keywords:** distraction osteogenesis, METTL3, angiogenesis, PI3K/AKT signaling pathway, endothelial progenitor cells

## Abstract

Distraction osteogenesis (DO) is used to treat large bone defects in the field of oral and maxillofacial surgery. Successful DO-mediated bone regeneration is dependent upon angiogenesis, and endothelial progenitor cells (EPCs) are key mediators of angiogenic processes. The *N6*-methyladenosine (m^6^A) methyltransferase has been identified as an important regulator of diverse biological processes, but its role in EPC-mediated angiogenesis during DO remains to be clarified. In the present study, we found that the level of m^6^A modification was significantly elevated during the process of DO and that it was also increased in the context of EPC angiogenesis under hypoxic conditions, which was characterized by increased METTL3 levels. After knocking down METTL3 in EPCs, m^6^A RNA methylation, proliferation, tube formation, migration, and chicken embryo chorioallantoic membrane (CAM) angiogenic activity were inhibited, whereas the opposite was observed upon the overexpression of METTL3. Mechanistically, METTL3 silencing reduced the levels of VEGF and PI3Kp110 as well as the phosphorylation of AKT, whereas METTL3 overexpression reduced these levels. SC79-mediated AKT phosphorylation was also able to restore the angiogenic capabilities of METTL3-deficient EPCs *in vitro* and *ex vivo*. *In vivo*, METTL3-overexpressing EPCs were additionally transplanted into the DO callus, significantly enhancing bone regeneration as evidenced by improved radiological and histological manifestations in a canine mandibular DO model after consolidation over a 4-week period. Overall, these results indicate that METTL3 accelerates bone regeneration during DO by enhancing EPC angiogenesis via the PI3K/AKT pathway.

## Introduction

Effectively and efficiently treating mandibular defects remains a major clinical challenge. Distraction osteogenesis (DO) is a surgical procedure wherein large bone defects arising as a consequence of injury, congenital malformation, or tumor excision can be repaired (Earley and Butts, 2014; Hatefi et al., 2019). Distraction osteogenesis induces robust regenerative activity conducive to osteogenesis between two surgical osteotomy sites through a process of gradual distraction (Runyan and Gabrick, 2017). Distraction osteogenesis is, however, a prolonged process associated with significant discomfort and high complication rates, limiting its more widespread clinical implementation (McCarthy et al., 2002). The mechanisms governing DO-related regeneration also remain to be clarified, and further research aimed at understanding these underlying molecular mechanisms may aid in shortening the DO-related treatment period, potentially improving the clinical utility of this procedure.

Osteogenesis is closely linked to angiogenesis in the context of bone development (Kusumbe et al., 2014; Sivaraj and Adams, 2016), and such angiogenesis has been found to be robust during the distraction phase of DO (Fang et al., 2005; Jacobsen et al., 2008). Insufficient vascularization is thought to be a primary cause of delayed union or non-union during bone repair (Deshpande et al., 2015; Stegen et al., 2015), and endothelial progenitor cells (EPCs) play a vital role in angiogenesis and vasculogenesis, homing to sites of tissue regeneration and remodeling and therein supporting these important processes (George et al., 2011). Endothelial progenitor cells also participate in DO and bone fracture (BF) regeneration (Lee et al., 2008; Atesok et al., 2010), potentially by facilitating the recruitment, proliferation, migration, and activity of skeletal progenitor cells (Han and Hsieh, 2014; Wen et al., 2016). Consequently, EPCs play an essential role by coupling angiogenesis and osteogenesis, and it is critical that the mechanistic role of EPCs in the context of DO-related angiogenesis be clarified.

*N*6-methyladenosine (m^6^A) is an epigenetic mRNA modification that is highly conserved, reversible, and prevalent in eukaryotic cells wherein it can influence RNA stability and processing (Frye et al., 2018; Wu et al., 2018). This dynamic methylation process is thought to be vital to the epigenetic regulation of mammalian cells (Zheng et al., 2013). M^6^A methylation-related enzymes include RNA methyltransferases, demethylases, and readers. Methyltransferase-like 3 (METTL3), together with its auxiliary partners METTL14 and WTAP, forms a methyltransferase complex to function as an m^6^A reader. These modifications can be erased by the demethylases FTO and ALKBH5. In addition, m^6^A reader proteins with a YT521-B homology (YTH) domain, including YTHDF1, YTHDF2, YTHDF3, YTHDC1, and YTHDC2, can specifically bind to m^6^A residues and modulate RNA processing accordingly (Liu et al., 2014; Tong et al., 2018; Lan et al., 2019; Shi et al., 2019). Emerging evidence suggests that m^6^A modifications play critical roles in biological processes including metabolism, embryogenesis, and development (Meyer and Jaffrey, 2014). METTL3 was the first methyltransferase identified as being linked to the process of m^6^A modification, forming the catalytic core of the associated modification complex (Zeng et al., 2020). It additionally functions as a regulator of cellular proliferation, migration, apoptosis, differentiation, metabolism, cell cycle progression, and innate immune/inflammatory functionality (Liu et al., 2020). In recent studies, METTL3 has been shown to promote the translation of a range of genes in the context of development or carcinogenesis (Deng et al., 2018; Frye et al., 2018), and to further sustain the self-renewal, migration, and stem-like properties of lung cancer cells (Lin et al., 2016; Li et al., 2019). METTL3 was also identified as a critical regulator of vasculogenesis in hematopoietic stem cells and progenitor cells during embryogenesis (Zhang et al., 2017). How these m^6^A regulators influence EPC-related angiogenesis in the context of DO, however, has not been established to date. The present study thus sought to explore the impact of m^6^A methylation on EPC angiogenesis and to clarify its effects on DO-mediated bone regeneration.

## Materials and Methods

### Mandibular Distraction Osteogenesis and Bone Fracture Model Establishment

**FIGURE 1:**
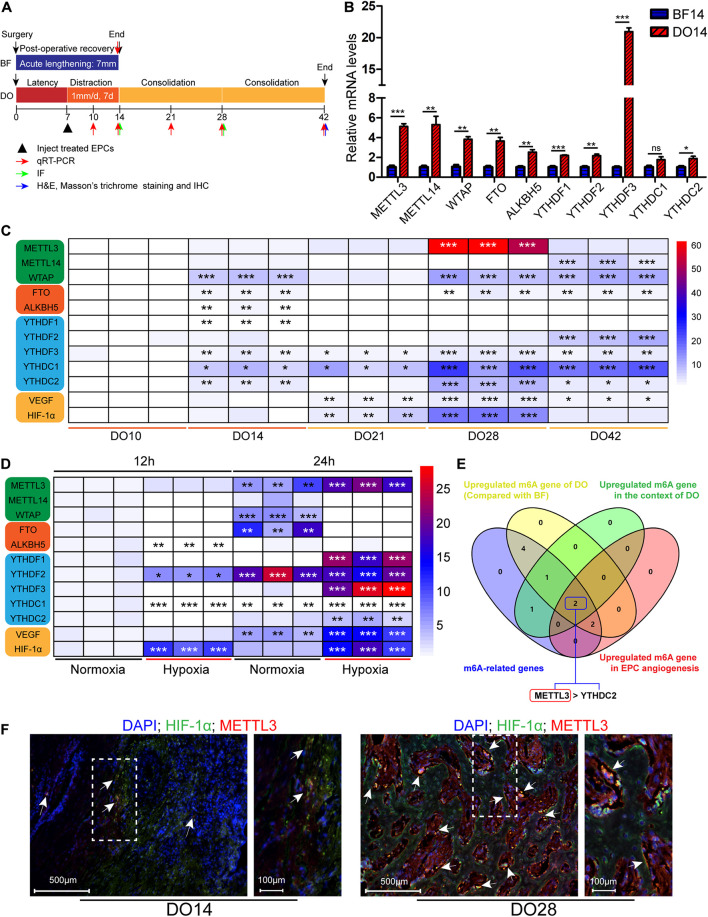
Methyltransferase-like 3 (METTL3) expression is elevated in endothelial progenitor cell (EPC) angiogenic processes related to distraction osteogenesis (DO) regeneration. **(A)** Schematic overview of the canine DO and BF model employed in this study. **(B)** quantitative real-time polymerase chain reaction (qRT-PCR) results revealed that m^6^A methylation was increased in the DO callus tissue relative to the bone fracture (BF) model (*n* = 3 per group). **(C)** Heatmap of qRT-PCR results demonstrating the expression of N6-methyladenosine (m^6^A)-related genes during different stages of DO (*n* = 3/group). **(D)** Heatmap of qRT-PCR results in the context of EPC angiogenesis. Endothelial progenitor cells were cultured under hypoxic conditions *in vitro* to mimic the DO microenvironment. N6-methyladenosine and angiogenic cytokine expression levels were measured following culture for 24 h (*n* = 3/group). **(E)** Venn diagram demonstrating m^6^A-related gene overlap. *METTL3* and *YTHDC2* were specifically identified, and *METTL3* was selected for further study owing to its high expression level. **(F)** IF staining for METTL3 and HIF-1α on DO14 and DO28. This analysis revealed that METTL3 was expressed on DO14 (white arrow) with gradual increases at later time points, and HIF-1α co-expression was also evident (*n* = 3/group). Scale bars: 500 and 100 μm. ^ns^*P* > 0.05, *P < 0.05, **P < 0.01, ***P < 0.001. One-way ANOVA with Tukey’s *post hoc test*. Experiments were repeated in triplicate.

Beagle dogs (*n* = 24; male, 1-year-old; 15–20 kg) were obtained from the Experimental Animal Center of Guangxi Medical University (Nanning, China). A mandibular DO model was established as in prior reports (Jiang et al., 2021). Briefly, animals were anesthetized via the intraperitoneal injection of pentobarbital (1 mg/kg) and xylazine (2 mg/kg) and underwent a right mandible osteotomy (Supplementary Figures 1A,B), with the distal and proximal segments being fixed using an internal distraction fixator (Cibei, China) (Supplementary Figure 1C). The skin was then closed using 4/0 polyglactin absorbable sutures (Supplementary Figure 1D). Following a 7-day latency phase, distraction was initiated for 7 days (1 mm twice per day), followed by the consolidation period. At appropriate time points including the middle (DO10) or end (DO14) of distraction, and after 1, 2, or 4 weeks of consolidation (DO21, DO28, and DO42), animals were euthanized via pentobarbital sodium overdose (Figure 1A). In the mandibular BF group, animals also underwent an osteotomy conducted in the same location, and after osteotomy the operating area was acutely lengthened to 7 mm and fixed with a titanium plate and nail (Cibei, China) (Supplementary Figures 1E,F). After 14 days of regeneration, the BF calluses were collected. The institutional Animal Care and Use Committee of Guangxi Medical University approved all animal studies described herein (No. 202101002).

### Immunofluorescence Analysis

Samples of DO callus collected on DO14 and DO28 (*n* = 3 each) were fixed using 4% PFA, decalcified over a 4-week period in a 10% EDTA solution, paraffin-embedded, cut with a rotary microtome (RM2255, Leica, Germany) to yield 4-μm sections that were permeabilized for 30 min with 0.5% Triton X-100, washed with PBS, and blocked for 30 min with 5% goat serum. Callus sections were probed with anti-METTL3 (1:100, Cat. ab195352, Abcam, United States) and anti-HIF-1α (1:100, MA1-1651B, ThermoFisher, United States), and EPCs were probed overnight with antibodies recognizing CD133, CD34, and VEGFR2 (Invitrogen, United States) at 4°C. The secondary anti-rabbit IgG (HS121-01, TransGen, China) was used to treat samples for 1 h. Finally, nuclei were stained with DAPI before imaging with an Intelligent Full-Automatic Fluorescence Microscopy Imaging System (Invitrogen^TM^ EVOS^TM^ FL Auto 2, Thermo Scientific, United States).

### Canine Endothelial Progenitor Cells Isolation, Cultivation, and Identification

Endothelial progenitor cells from three different canines were isolated and grown according to our previous study (Zhu et al., 2021). Briefly, mononuclear cells (MNCs) were obtained via density gradient centrifugation using a Canine Bone Marrow mononuclear cell isolation kit (P6090, Solarbio, China), after which cells were resuspended in complete endothelial growth medium-2 (EGM-2; Lonza, United States), and seeded in fibronectin-coated T25 flasks at 37°C in a 5% CO_2_ humidified incubator. When cells were 80–90% confluent, they were split. After two passages, cells were used for subsequent experimentation. Immunofluorescent staining was used to assess EPC phenotypes, which the characteristics of these cells were examined through assays of FITC-UEA-1 binding and Dil-ac-LDL uptake.

### Lentiviral Transduction and Pathway Activation

Methyltransferase-like 3 effects on EPC angiogenic activity were examined using METTL3-shRNA and overexpression (OE) lentiviral vectors. Lentivirus-mediated OE, negative control, and silencing vectors were generated by cloning three shRNA sequences targeting cfa-METTL3 (Table 1) into the hU6-MCS-CBh-gcGFP-IRES-puromycin vector. For METTL3-OE vectors, the full-length cfa-METTL3 (XM_532627.6) sequence was cloned into a Ubi-MCS-3FLAG-SV40-EGFP-IRES-puromycin lentivirus vector. Viruses were utilized to infect cells at an MOI of 50 based on the manufacturer’s instructions (GeneChem, China). After transduction, 1 μg/mL puromycin was added per well to select for infected cells. To detect the pathway regulated by METTL3, these cells were treated with the AKT activator SC79 (HY-18749, MCE, China) at 4 μg/ml for 4 h with control shRNA or METTL3 inhibitor treatment.

**TABLE 1:** The sequence of lentivirus.

Lentivirus	Sequence
NC-shRNA	TTCTCCGAACGTGTCACGT
METTL3-shRNA1	GGGCACCTGGATCTACGAAAT
METTL3-shRNA2	CCGCAGTTCCTGAACTAGCTA
METTL3-shRNA3	ATGCTGATCATTCCAAGCTTT

*NC, negative control; shRNA, short hairpin RNAs.*

### RNA Extraction and Quantitative Real-Time PCR

TRIzol (Invitrogen, CA, United States) was used to isolate total RNA from DO, BF callus, and cell samples as per provided directions. A RevertAid First Strand cDNA Synthesis Kit (Invitrogen) was then used to prepare cDNA from 5 μg total RNA samples, with subsequent qRT-PCR assays being performed using 2 μl of cDNA (5 ng/μl) in a 20 μl volume containing with 2 × PowerUp SYBR Green Master Mix (Invitrogen) with a QuantStudio-5 system (Applied Biosystems, United States). Primers used herein are shown in Table 2. Relative gene expression was measured via the 2^–^^△△^^Ct^ approach, with GAPDH being used for normalization purposes. All data were visualized by RStudio.

**TABLE 2:** Primer sequences.

Gene	Forward (5′–3′)	Reverse (5′–3′)
*METTL3*	GCCGTACAGGTCACTGG TTGAAC	TCTTGCGAGTGCCAGGAGA TAGTC
*METTL14*	TTGGACCTTGGAAGAGTGTG TTTACG	TTGTTCTCTGGAAGACAGCC TTTGG
*WTAP*	CGACTAGCAACCAAGGAGCA AGAG	AAACAAGTTGATCGCTGGGT CTACC
*FTO*	GGAAGGACTGTGGAAGAAG ATGGAAG	GTGTAGTGAGCAAGGCAAG GATGG
*ALKBH5*	CCATCGTGTCCGTGTCCT TCTTC	CAGCAGCATATCCACTGAG CACAG
*YTHDF1*	GGCATGGTGGGTCTGAAG ATTGG	GAGGTTGGCTTTGAAACTG GCATATTG
*YTHDF2*	GGCAGTGGGTTCGGTCAT AATGG	TGGGTGAGGTTCTGAAGGA GTAGATC
*YTHDF3*	CAGCCTTGAGCAGCAGTG GTATG	GGGTTTAAGTTTCGGTTGAGG TTTGG
*YTHDC1*	TGGCAGCAGTGGTTCTTCT GATG	TCCTCCTCCTCCTCCTCT TCTCC
*YTHDC2*	GAAGAGAAACAACAAAGCAC CCTTACG	AAGACAGCATCACCGCCA TCATC
*VEGF*	TCCACCATGCCAAG TGGT	CCATGAACTTCACCA CTTCG
*HIF-1*α	TTGCTCATCAGTTGCCA CTTCC	GCAATTCATCTGTGCCTTC ATTTC
*GAPDH*	ATTCCACGGCACAGTCA AGG	ACAT ACTCA GCACCAGCATC

### Western Blotting

On day 3 post-transduction, EPCs were lysed with RIPA buffer containing the Halt^TM^ Protease and Phosphatase Inhibitor Single-Use Cocktail (Thermo, United States). Lysate supernatants were collected after a 15 min spin at 12,000 × *g* at 4°C, and a bicinchoninic acid (BCA) Protein Assay kit (Beyotime) was used to quantify protein levels in these samples. Samples of protein (25 μg) were then separated via 10% SDS-PAGE and transferred to PVDF membranes (Millipore, MA, United States). Blots were washed thrice with TBST, blocked for 1 h with 5% non-fat milk, and incubated with antibodies specific for VEGF (1:1,000, MA1–16629, Thermo), METTL3 (1:1,000), PI3Kp110 (1:1,000, ab32569, Abcam), anti-AKT (1:1,000, 9272, CST), anti-phosphorylated (p-AKT, 1:500, 9272, CST), or GAPDH (1:1,000, AG019, Beyotime) overnight at 4°C. Blots were then probed with secondary anti-mouse (1:1,000, A0216, Beyotime) or anti-rabbit (1:1,000, A0208, Beyotime) antibodies for 2 h, after which the ECL plus (P0018M, Beyotime, China) reagent was used to detect protein bands. GAPDH served as a normalization control.

### *N6*-Methyladenosine RNA Methylation Quantification

An EpiQuik^TM^ m^6^A RNA Methylation Quantification Kit (Epigentek, United States) was used based on provided instructions to measure m^6^A methylation levels. Briefly, an RNA high binding solution was used to bind 200 ng of total RNA samples, after which capture and detection antibodies were used to detect m^6^A modifications. The levels of m^6^A in individual samples were then quantified by adding enhancer color development solutions and assessing absorbance at 450 nm using a microplate spectrophotometer (Infinite MFlex, TECAN).

### Cell Proliferation Assay

A Cell Counting Kit-8 (CCK-8, Dojindo, Japan) was used to measure cell proliferation. Briefly, 5 × 10^3^ EPCs were added per well of a 96-well plate. After 48 h, 10 μl of CCK-8 solution was added per well. Following a 3 h incubation at 37°C, absorbance at 450 nm was measured via microplate reader (Infinite MFlex, TECAN). Cells from three different canine lines were used (*n* = 3 per line for each group).

### Tube Formation Assay

Matrigel was used to measure EPC tube formation as reported previously (Zhu et al., 2021). Briefly, Matrigel (Corning Co. Ltd., United States) was thawed at 4°C overnight, after which 10 μl of gel was transferred to a cold μ-Slide plate using a chilled micropipette. Matrigel solidification was then achieved via incubation for 45 min at 37°C, after which treated EPCs (1.5 × 10^4^ cells/well, *n* = 3 per cell line; *n* = 3 cell lines per group) were added to the Matrigel. Tube formation was then assessed following a 6 h incubation at 37°C, with three random microscopic fields of view per sample being assessed. The angiogenic ability in each field was quantified using the Image J software.

### Cell Migration Assay

Cell migration was evaluated using Transwell and wound healing assays (*n* = 3 per cell line; *n* = 3 cell lines per group). For the Transwell assay, treated EPCs were resuspended at 2 × 10^4^ cells/ml in serum-free media, and a 500 μl volume was added to the upper portion of a Transwell assay insert (8.0 μm, Corning, NY, United States) in a 24-well plate, with 750 μl of complete media being added into the lower chamber. Following a 48 h incubation, a cotton swab was used to remove non-migratory cells, while those cells that did migrate were fixed for 20 min with 4% PFA, stained for 30 min with 0.5% crystal violet, and counted under an inverted microscope (100×; 5 fields of view per sample). For wound healing assays, treated EPCs were added to 6-well plates (1.5 × 10^5^ cells/well) and allowed to grow until 70–80% confluent, at which time a sterile 200 μl pipette tip was used to generate a straight wound in the monolayer. Cells were then cultured in serum-free media, and wounds were imaged via an inverted microscope at 0 and 24 h, with ImageJ being used to quantify cell migration.

### Chicken Chorioallantoic Membrane Assay

A chicken embryo chorioallantoic membrane (CAM) assay was conducted as in prior reports (Jiang et al., 2021). Briefly, fertilized chicken eggs were cultured at 37°C for 7 days in a 65% humidity environment, after which the air chamber position was marked on the eggshell, and a portion of the shell was removed to reveal the CAM, which was treated via the injection of treated EPCs (*n* = 3 per cell line; *n* = 3 cell lines per group). Following an additional 3-day incubation, the CAM was imaged with a stereomicroscope (SMZ745T, Nikon, Japan). The AngioTool software was then used to measure CAM vascular density.

### Bioinformatics Analyses

Target genes regulated by METTL3 were predicted using m6A2Target.^1^ KOBAS 3.0^2^ was used to conduct a gene ontology (GO) analysis and to assess angiogenesis-related genes. These genes were reimported into KOBAS 3.0 to conduct a Kyoto Encyclopedia of Genes and Genomes (KEGG) pathway analysis. All data were visualized using RStudio.

### Micro-Computed Tomography

Distracted mandible samples were harvested after a 4-week consolidation period (DO42). Micro-CT scanning (Latheta LCT-200, Hitachi Aloka Medical, Nagasaki, Japan) was performed to quantitatively evaluate bone regeneration within the distraction zone with an 80 mm scan view and a resolution of 40 × 40μm. Thereafter, a three-dimensional (3D) reconstruction of the lengthened mandible was generated using the VGStudio Max 2.2 software (Volume Graphics GmbH, Germany). The bone mineral density (BMD) of the regenerated bone was assessed using Latheta V3.61b. Other parameters, including bone volume/tissue volume (BV/TV), trabecular separation (Tb. Sp), trabecular number (Tb. N), and trabecular thickness (Tb. Th), were analyzed using the VGStudio Max 2.2 software.

### Histological and Immunohistochemical Staining

Following the 4-week consolidation period, DO callus samples (*n* = 3/group) were collected, fixed at room temperature with 4% PFA, decalcified for 8 weeks in 10% EDTA (Solarbio, China), dehydrated using an ethanol gradient, paraffin-embedded, and cut with a rotary microtome (RM2255, Leica) into 4-μm longitudinal sections. Sections were treated with xylene for deparaffinization, rehydrated with an ethanol gradient, and subjected to Masson’s trichrome or H&E staining for histological analyses. IHC staining was conducted by treating samples for 20 min with sodium citrate buffer (0.01 mol/L, pH 6.0) at 95°C to facilitate antigen retrieval, followed by a 10 min treatment with endogenous peroxidase blocker (#PV-6000, ZSGB-Bio, China). Sections were then probed for 1 h with anti-OCN (1:100, #orb348959, biorbyt, British) at 37°C, followed by secondary antibody (#PV-6000, ZSGB-Bio) incubation for 20 min at room temperature. A DAB kit (#ZLI-9018, ZSGB-Bio) was then used for protein detection, followed by hematoxylin counterstaining.

### Statistical Analysis

SPSS 23.0 was used for all statistical testing. Data are given as means ± standard deviation (SD) and were analyzed using Student’s *t*-tests between two groups and one-way analysis of variance (ANOVAs) with Tukey’s *post hoc test* among multiple groups, with *P* < 0.05 as the significance threshold. Experiments were repeated in triplicate, with representative results being shown.

## Results

### Methyltransferase-Like 3 Upregulation Occurs in the Context of Distraction Osteogenesis and Endothelial Progenitor Cell Angiogenesis

To establish whether m^6^A modifications are associated with DO-related regeneration, we established canine mandibular DO and BF models (Figure 1A), and monitored the expression of major m^6^A methylation-related modifying enzymes within the DO and BF callus regions. qRT-PCR revealed that almost all m^6^A-related genes were upregulated in DO14 callus samples as relative to BF14 samples (Figure 1B). To illustrate the dynamic changes that occur in the context of DO, we detected m^6^A-related gene expression patterns associated with different phases in DO, revealing that *METTL3*, *WTAP*, *YTHDC1*, and *YTHDC2* were expressed at significantly higher levels after 2 weeks of consolidation (DO28) relative to DO10 samples, as were levels of the angiogenesis-related cytokine *VEGF* and hypoxia-related *HIF-1*α (Figure 1C). We thus speculated that alterations in RNA m^6^A methylation status may be closely related to the high degree of regeneration that occurs during DO.

Endothelial progenitor cells can enhance bone regeneration and shorten the treatment duration of DO. As such, we next isolated EPCs from canine bone marrow and identified these cells based on their morphology (Supplementary Figures 2A,B), angiogenic ability (Supplementary Figure 2C) surface markers (Supplementary Figures 2D,E), and via Dil-ac-LDL uptake and FITC-UEA-1 binding assays (Supplementary Figure 2F). To simulate the EPC angiogenesis conditions during DO *in vitro*, EPCs were cultured in a hypoxic (1% O_2_) incubator, and we found that *METTL3*, *YTHDF1*, *YTHDF3*, and *YTHDC2* were significantly upregulated in EPCs in response to hypoxia at the mRNA level as compared with cells cultured under normoxic conditions (Figure 1D). *VEGF* and *HIF-1*α were also upregulated in this context (Figure 1D). These results imply that METTL3 functions as the primary m^6^A writer that is involved in the regulation of DO given the marked changes in its observed expression (Figure 1E). Additionally, immunofluorescent staining revealed that METTL3 underwent gradual upregulation in callus tissues during the consolidation phase and was co-expressed with HIF-1α (Figure 1F). Together, these findings suggest that METTL3 may regulate EPC angiogenesis in the context of DO.

### Methyltransferase-Like 3 Regulates Endothelial Progenitor Cells Proliferation, Migration, and Tube Formation

**FIGURE 2:**
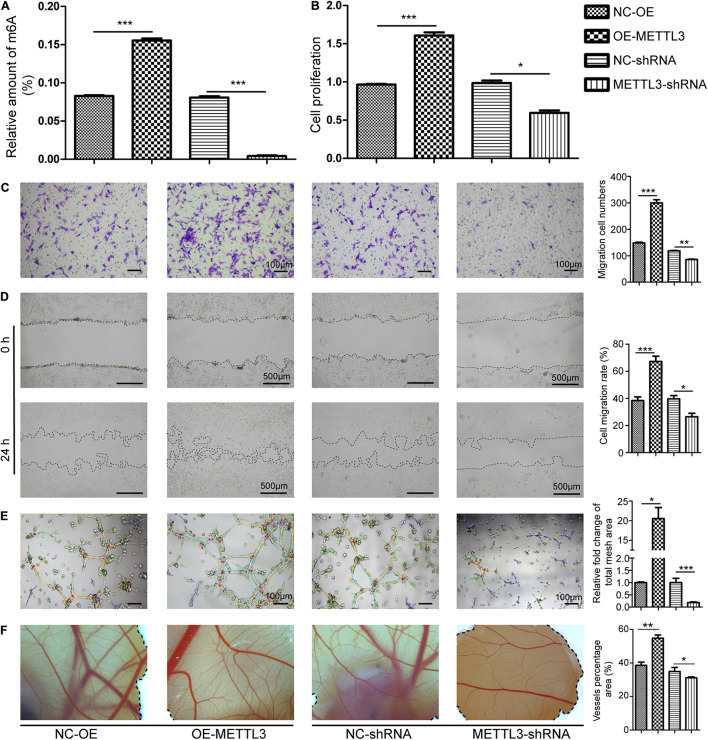
Methyltransferase-like 3 impacts m^6^A methylation and EPC-mediated angiogenesis. **(A)** Total m^6^A RNA methylation levels were quantified in EPCs following knockdown or overexpression. **(B)** Endothelial progenitor cell proliferation was measured via CCK-8 assay. METTL3 overexpression enhanced cellular proliferation, while silencing impairs such proliferation. **(C–F)** *In vitro* EPC angiogenesis was measured through Transwell **(C)**, Wound healing **(D)**, and Tube formation assays **(E)**. Scale bars: 100 and 500 μm. **(F)** METTL3 affects *ex vivo* angiogenesis. CAM assays revealed that METTL3 overexpression significantly increased vascular density, while METTL3 inhibition had the opposite effect. NC-OE and OE-METTL3 groups or NC-shRNA and METTL3-shRNA groups were compared via Student’s *t*-tests. *P < 0.05, **P < 0.01, ***P < 0.001. All cell experiments were conducted in triplicates using three individual cell lines.

To directly assess the functional role of METTL3 in the context of EPC angiogenesis, overexpression negative control (NC-OE), METTL3 overexpression (OE-METTL3), shRNA negative control (NC-shRNA), and three METTL3-specific shRNA constructs were designed. qRT-PCR and Western Blotting (WB) were used to confirm the successful overexpression (Supplementary Figures 3A,B) or knockdown of this gene in EPCs (Supplementary Figures 3C,D). METTL3 overexpression markedly increased m^6^A levels in cells, while METTL3 knockdown significantly reduced m^6^A levels (Figure 2A). Next, we analyzed the functional effects of METTL3 on the proliferation, tube formation, and migratory activity of EPCs. When METTL3 was overexpressed in these cells, they exhibited significantly enhanced proliferation, whereas METTL3 silencing had the opposite impact (Figure 2B). Moreover, we found that METTL3 overexpression was associated with enhanced EPC migration in Transwell (Figure 2C) and wound healing (Figure 2D) assays, whereas METTL3 inhibition suppressed these activities. Tube formation is a key step in the process of angiogenesis. EPC tube formation was augmented by overexpressing METTL3 (Figure 2E), while this ability was disrupted following METTL3 knockdown as evidenced by reduced capillary-like structure formation (Figure 2E). Three days after injecting treated EPCs into a CAM model, embryos injected with METTL3-overexpressing cells exhibited increased vessel density, whereas reduced branching was evident following the injection of METTL3 knockdown EPCs, and vessel density was lower in these samples (Figure 2F). Together, these data suggested that METTL3 expression could effectively promote EPC angiogenesis, highlighting the potential regulatory roles of this gene in this DO-related regenerative context.

### Methyltransferase-Like 3 Regulates Endothelial Progenitor Cell Angiogenesis via the PI3K/Activate Protein Kinase B Signaling Pathway

**FIGURE 3:**
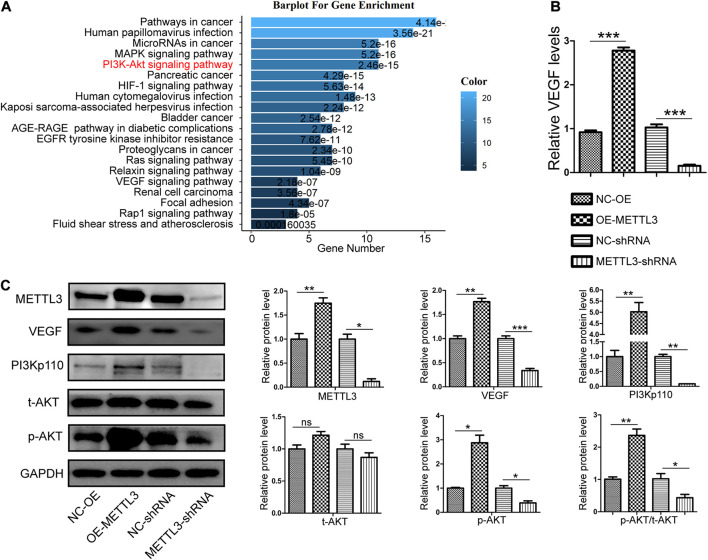
Methyltransferase-like 3 enhances PI3K/AKT signaling pathway activation and angiogenic cytokine production. **(A)** Gene Ontology enrichment analysis of the angiogenic targets of METTL3. **(B)** Following METTL3 knockdown or overexpression, the mRNA levels of the angiogenic cytokine *VEGF* were significantly altered. **(C)** PI3K/AKT signaling pathway and angiogenesis-related proteins including PI3Kp110, AKT, p-AKT, and VEGF were assessed via Western blotting in EPCs, with ImageJ being used for analysis. Analyses were conducted using data from three independent experiments. NC-OE and OE-METTL3 groups or NC-shRNA and METTL3-shRNA groups were compared *via* Student’s *t*-tests. ^ns^*P* > 0.05, *P < 0.05, **P < 0.01, ***P < 0.001; *n* = 3 in each independent experiments.

To detect downstream signaling molecules that may be activated by METTL3, we used m6A2Target^3^ to search for METTL3 target genes (Supplementary Table 1). Gene ontology (GO) analyses were then performed to search for targets involved in angiogenesis (Supplementary Tables 2,3) and to predict the possible signaling pathways that are regulated by METTL3. These angiogenesis-related pathways included the MAPK, PI3K/AKT, HIF-1α, Ras, Relaxin, VEGF, and Rap1 signaling pathways (Figure 3A). To explore the mechanisms whereby METTL3 promotes canine EPC angiogenesis, we next analyzed the PI3K/AKT pathway given its well-documented importance in angiogenesis (Karar and Maity, 2011) and it is directly downstream of VEGF (Claesson-Welsh and Welsh, 2013). qRT-PCR analyses revealed that METTL3 overexpression was associated with significant increases in the expression of *VEGF*, whereas the opposite was observed following METTL3 knockdown (Figure 3B). WB results further revealed that METTL3 overexpression was associated with higher levels of PI3Kp110 and p-AKT in EPCs, while knockdown METTL3 reduced the levels of these key signaling proteins (Figure 3C). Similarly, VEGF protein levels were, respectively, enhanced and suppressed by METTL3 overexpression and depletion (Figure 3C). Together, these data suggested that METTL3 may modulate the PI3K/AKT pathway to control EPC angiogenesis.

### The PI3K/Activate Protein Kinase B Pathway Activator SC79 Rescues Defective Angiogenesis

**FIGURE 4:**
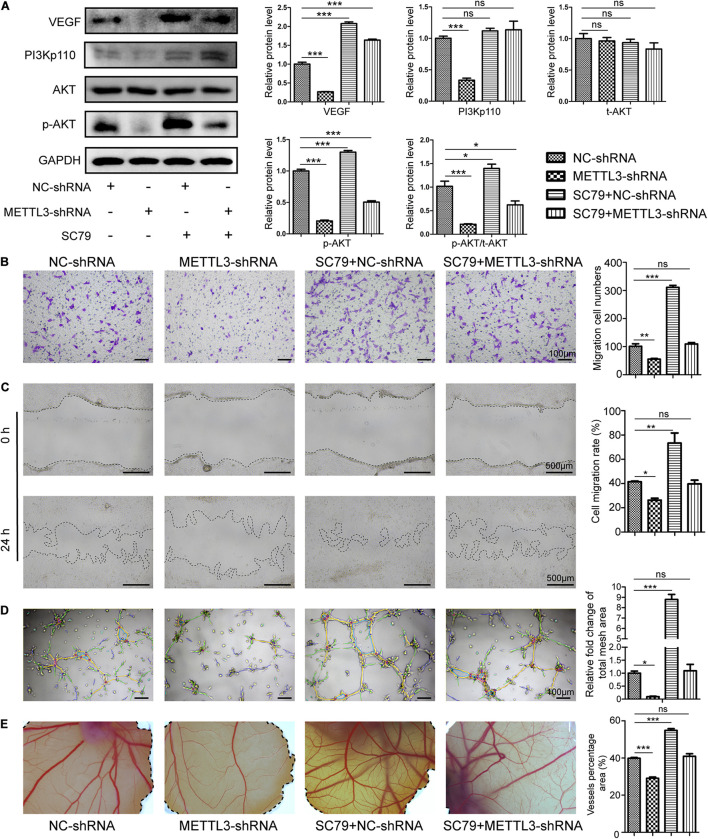
Methyltransferase-like 3 regulates EPC angiogenesis via activating the PI3K/AKT pathway. **(A)** Western blotting analysis of PI3K/AKT pathway proteins in negative control and METTL3-deficient EPCs which were treated with SC79. **(B–E)** Migration assay **(B,C)**, Tube formation **(D)**, and CAM angiogenesis assays **(E)** were conducted using negative control and METTL3-deficient cells after SC79 treatment. Scale bars: 100 and 500 μm. These results indicated that injection with METTL3-knockdown EPCs was sufficient to reduce EPC angiogenic activity, whereas SC79 was able to reverse such impairment. Data are means ± SD and were compared via one-way ANOVA with Tukey’s *post hoc* test. ^ns^*P* > 0.05, *P < 0.05, **P < 0.01, ***P < 0.001 vs. NC-shRNA; *n* = 3 per group, experiments were repeated in triplicate.

To further verify that the PI3K/AKT pathway is essential for METTL3-regulated angiogenesis, we use the SC79 to augment Akt phosphorylation in the negative control and METTL3-deficient EPCs. WB results indicated that p-Akt protein levels increased markedly in the presence of this AKT activator (Figure 4A) and led to a substantial increase in migration (Figures 4B,C) and tube formation (Figure 4D) *in vitro*, and CAM angiogenesis *ex vivo* (Figure 4E). Moreover, METTL3-shRNA treatment decreased VEGF, PI3K, and p-AKT protein expression and associated angiogenesis (Figure 4A). Moreover, SC79 co-treatment with METTL3-shRNA rescued the expression of p-AKT (Figure 4A) and the effect on angiogenesis (Figures 4B–E). Overall, our results suggested that the vascular effects of METTL3 in EPC are mediated in part by its ability to regulate the PI3K/AKT pathway.

### Methyltransferase-Like 3 Promotes Distraction Osteogenesis-Related Ossification *in vivo*

**FIGURE 5:**
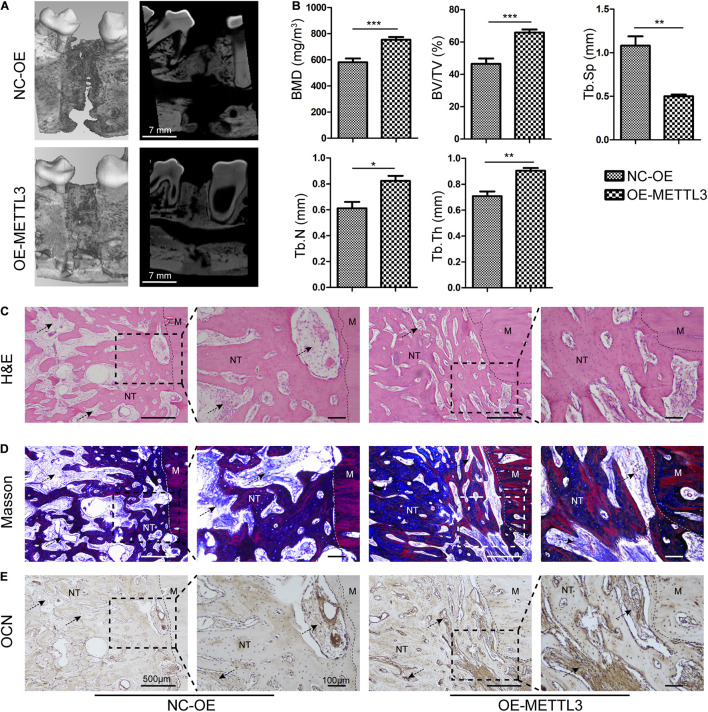
Methyltransferase-like 3 regulates angiogenesis and DO regeneration responses. **(A,B)** Representative 3D and longitudinal images **(A)** and quantitative analysis **(B)** of micro-CT data, including bone mineral density (BMD), bone volume/tissue volume (BV/TV), trabecular separation (Tb. Sp), trabecular number (Tb. N), and trabecular thickness (Tb. Th) of the mandibular distraction area after 4 weeks of consolidation. **(C,D)** For histological analyses, H&E **(C)** and Masson’s trichrome **(D)** staining of the MDO callus were performed in the different groups. **(E)** IHC assessment of OCN in the NC-OE and OE-METTL3 groups. NC-OE and OE-METTL3 groups were compared via Student’s *t*-tests. Arrows, extracellular matrix. M, Mandible, NT, Newly formed trabecula. Scale bars: 100 and 500 μm. *P < 0.05, **P < 0.01, ***P < 0.001 vs. NC-OE; *n* = 3 canine in each group.

To further explore the effects of METTL3 on DO regeneration, 1 × 10^7^ autologous EPCs overexpressing METTL3 were injected into the distraction gap at the start of the distraction period. Micro-computed tomography (μCT) analysis of trabecular bone regeneration revealed that the cortical bone within the distraction area to be nearly continuous in the METTL3-OE group, with abundant callus formation. In contrast, the bone regeneration in the control groups remained unsatisfactory, with discontinuous cortical bone formation (Figure 5A). The bone mineral density (BMD) and bone volume/tissue volume ratio (BV/TV) were significantly elevated in the METTL3-OE group. Moreover, METTL3 overexpression increased the trabecular number (Tb. N), and trabecular thickness (Tb. Th), while reducing the trabecular separation (Tb. Sp) (Figure 5B). In addition, the newly formed canine mandibular bone was examined via H&E and Masson’s trichrome staining. Relative to the control DO group, calluses from the METTL3-OE group exhibited larger quantities of organized, mineralized bone tissue with lamellar morphology at 4 weeks of consolidation. Moreover, the demarcation between this new bone tissue and the normal mandible was barely evident in the METTL3 group (Figures 5C,D). Additionally, immunohistochemical staining results indicated that the levels of the osteogenesis-related protein OCN were significantly increased in OE group tissue, particularly in the ECM. The wide deposition was observed in the new bone trabecular tissue in the OE group (Figure 5E), consistent with the profound osteogenic activity of METTL3 in the canine DO model.

## Discussion

While DO can effectively repair large bone defects, its long consolidation phase and associated complications limit its widespread clinical implementation. Many studies have sought to shorten this consolidation phase through the use of growth factors (Makhdom and Hamdy, 2013), laser therapy (Sarmadi et al., 2019), hormones (Kang et al., 2017; Weng et al., 2019), and stem cell therapy (Jiang et al., 2021), but the mechanistic basis underlying this regenerative process has yet to be clarified. Studies of rats and rabbits are widely used when conducting mandibular DO research, whereas there have been fewer studies using large animal models. While rodent DO models are easier to implement, they are hampered by large discrepancies in mandibular size, morphology, and function relative to those of humans (Tee and Sun, 2015). As such, we selected canines as a model to explore the mechanisms governing mandibular DO regeneration in the present study.

The dysregulation of genes associated with the modification of interpretation of the m^6^A modification status of RNA molecules has recently been linked to RNA stability, transcription, nuclear transport, translation, and shearing (Wang et al., 2020). Such m^6^A modifications are thought to be directly relevant in the context of human health and disease (Shi et al., 2019). Bone formation in the context of DO is also extremely rapid, proceeding at a rate 4–8 times as fast as that observed in the context of rapid epiphysis bone formation in adolescents (Choi et al., 2002), with this rate being far faster than that normally observed in the context of fracture healing. To verify the rapid osteogenic rate during DO, we established DO and BF models to elucidate the unique modification patterns and their functional roles in the context of DO. The present study revealed that several m^6^A-related cytokines were significantly differentially expressed in these model systems, with *METTL3*, *WTAP*, *YTHDC1*, and *YTHDC2* being significantly upregulated during bone regeneration, suggesting that m^6^A methylation plays an important role in DO and promotes enhanced regeneration.

Angiogenesis and associated vascular responses are regulated by many different growth factors in the context of DO-related repair and regeneration (Donneys et al., 2012). In a rat model of mandibular DO, for example, EPCs home to ischemic tissue gaps and enhance local angiogenesis, bone formation, and mineralization during the consolidation stage (Cetrulo et al., 2005), in addition to shortening the duration of DO-related bone regeneration (Davidson et al., 2011). Endothelial progenitor cells are essential regulators of DO-related osteogenesis, and hypoxia is an important driver of angiogenesis, osteogenesis, and associated processes (Melero-Martin et al., 2008; Ransom et al., 2018). We, therefore, cultured EPCs in a hypoxic microenvironment to simulate physiological conditions associated with DO-related angiogenesis in an effort to confirm the role of m^6^A methylation in these processes. These analyses indicated that METTL3 expression was maximally increased under these conditions. Notably, hypoxia can induce METTL3 upregulation in the context of pathological angiogenesis, underscoring the relevance of m^6^A methylation as a regulator of vascular homeostasis in previous studies (Yao et al., 2020). Furthermore, a hypoxic microenvironment exists within the distraction gap, as evidenced by the high level of HIF-1α expression observed therein (Mori et al., 2006; Kumabe et al., 2020). To further confirm the role of METTL3 in these processes, we employed an immunofluorescent staining approach to detect METTL3 and HIF-1α levels in our model of DO. As expected, METTL3 was gradually upregulated in the DO callus in these animals, and was co-expressed with HIF-1α.

Notably, METTL3 is involved in a range of biological processes (Liu et al., 2020). Its expression patterns and functional roles during EPC angiogenesis, however, have yet to be reported. We found that knocking down METTL3 in EPCs reduced total RNA m^6^A methylation levels while also impairing EPC proliferation, migration, and tube formation. METTL3 overexpression, in contrast, promoted EPC angiogenesis. The chicken CAM structure is highly vascularized and is not susceptible to immunological rejection, making it an ideal model for *ex vivo* analyses of angiogenic processes (Rezzola et al., 2020). We also found that the overexpression or knockdown of METTL3 in EPCs was sufficient to alter their *ex vivo* angiogenic capabilities. Together, these data indicated that targeting m^6^A modifications via regulating METTL3 expression levels may be a promising approach to enhancing DO regeneration and EPC angiogenesis.

PI3K/AKT signaling is particularly important in the context of the regulation of angiogenesis, with prior studies having shown that the activation of this signaling pathway can enhance both osteogenesis and angiogenesis (Yang et al., 2019; Mi et al., 2020). Bioinformatics analyses further illustrated that the PI3K/AKT signaling plays an important role in m^6^A-related angiogenesis. Consistent with this, we determined that METTL3 overexpression enhanced the expression of VEGF and PI3Kp110 as well as the phosphorylation of AKT, whereas METTL3 knockdown had the opposite effect. Notably, the phosphorylation of AKT is essential for the PI3K/AKT pathway (Yudushkin, 2019). In order to firmly establish whether METTL3 modulates EPC angiogenesis via this pathway, SC79, an AKT phosphorylation activator, was used to reverse the impaired angiogenesis induced by METTL3 silencing. SC79 treatment was sufficient to restore AKT phosphorylation and did not influence the expression of PI3K. Interestingly, relative to the NC group, the VEGF and PI3K protein levels appeared to be increased in the co-treatment group. This may be because the PI3K/AKT pathway was able to enhance VEGF signaling pathway activity (Karar and Maity, 2011), and VEGF activates the PI3K/AKT pathway (Maity et al., 2000; Morales-Ruiz et al., 2000). Finally, the local injection of METTL3-overexpressing EPCs into the mandibular DO gap enhanced DO mineralization. Together, our data thus confirmed that METTL3 is an important regulator of DO-related regeneration and EPC angiogenesis, controlling these processes through mechanisms associated with m^6^A methylation.

Despite our promising results, the current study has several limitations. First, although we utilized a larger animal model to perform this DO research, the m^6^A-related gene database for canines remains relatively sparse, and much remains to be discovered. As such, future studies will need to employ m^6^A-seq, ribo-seq, and RNA-seq approaches in concert in order to more fully elucidate the mechanistic basis for DO. Second, we only assessed the therapeutic effects of METTL3 after a 4-week consolidation period. In the future, we will monitor the earlier phases of consolidation at 1- or 2-week time points to gain more comprehensive insights into the underlying histological changes.

**FIGURE 6:**
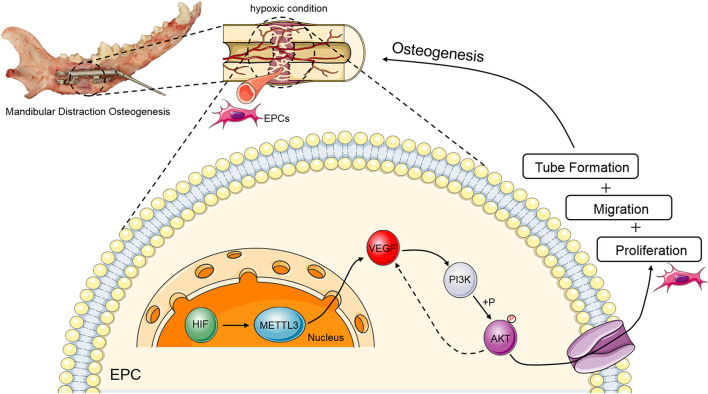
Schematic overview of the mechanisms whereby METTL3 controls PI3K/AKT signaling activity in the context of DO-related EPC angiogenesis. During DO-related tissue regeneration, the DO gap is a hypoxic environment. Upon upregulation, METTL3 can target VEGF to gradually increase and positively regulate PI3K activity and AKT phosphorylation, with such PI3K/AKT signaling partially strengthening the expression of VEGF, ultimately promoting EPC proliferation, migration, and tube formation during DO and thereby facilitating osteogenesis.

In summary, the results of the present study indicate that METTL3 improves the proliferation, migration, and tube formation activity of EPCs via the PI3K/AKT signaling pathway, indicating that METTL3 may play a critical role in the context of DO (Figure 6). Moreover, these results highlight a novel mechanism whereby m^6^A modifications regulate EPC-mediated angiogenesis during DO, suggesting a novel therapeutic approach to shortening the DO consolidation phase and accelerating bone regeneration in other pathological contexts.

## Data Availability Statement

The original contributions presented in the study are included in the article/Supplementary Material, further inquiries can be directed to the corresponding authors.

## Ethics Statement

The animal study was reviewed and approved by the animal study was reviewed and approved by the institutional Animal Care and Use Committee of Guangxi Medical University (No. 202101002).

## Author Contributions

WJ, TZ, and ZZ conducted experimentation on animals. PZ and FH conducted experiments *in vitro*. YL and LG carried out all statistical analysis, data curation, and formal analysis. FL and XA organized the pictures. PZ and WJ completed the manuscript. XH and NZ read and revised the manuscript. All authors conceived and designed the study, read, and approved the submission.

## Conflict of Interest

The authors declare that the research was conducted in the absence of any commercial or financial relationships that could be construed as a potential conflict of interest.

## Publisher’s Note

All claims expressed in this article are solely those of the authors and do not necessarily represent those of their affiliated organizations, or those of the publisher, the editors and the reviewers. Any product that may be evaluated in this article, or claim that may be made by its manufacturer, is not guaranteed or endorsed by the publisher.

## Footnotes

1
http://m6a2target.canceromics.org/#/home

2
http://kobas.cbi.pku.edu.cn/kobas3

3
http://m6a2target.canceromics.org/#/home

## Supplementary Material

Supplementary Figure 1 Canine mandibular DO and BF model procedure. **(A)** Mandible exposure. **(B)** Osteotomy. **(C)** Distraction device installation. **(D)** Skin suturing (DO group). **(E)** Titanium plate and nail installation following osteotomy (BF group). **(F)** Mandibular skin closure.

Supplementary Figure 2 Endothelial progenitor cells (EPC) Characterization. **(A–C)** Changes and tube formation for isolated EPCs were assessed over time. Following a 3-day culture period, a limited number of adherent cells were evident **(A)**, with limited proliferation **(B)**. The angiogenic activity of these EPCs was assessed via tube formation assay **(C)**. **(D–F)** Canine EPCs were identified by staining for CD133 (Red), CD34 (Red), and VEGFR2 (Green) on the cell surface **(D,E)**. Dil-ac-LDL uptake (Orange) and FITC-UEA-1 binding (Green) were assessed to gauge EPC functionality **(F)**.

Supplementary Figure 3 Methyltransferase-like 3 (METTL3) knockdown or overexpression in EPCs. **(A,B)** METTL3 overexpression was confirmed by qRT-PCR **(A)** and Western blotting **(B)**. **(C,D)** Relative EPC METTL3 mRNA **(C)** and protein **(D)** levels following NC-shRNA, METTL3-shRNA1, METTL3-shRNA2, or METTL3-shRNA3 transfection. Of these shRNAs, shRNA2 was the most efficacious and was therefore used for subsequent experiments. ^ns^*P* > 0.05, ^∗^*P* < 0.05, ^∗∗^*P* < 0.01, ^∗∗∗^*P* < 0.001; *n* = 3 in each independent experiments.

## Abbreviations

ALKBH5: a-ketoglutarate -dependent dioxygenase alkB homolog 5
AKT: activate protein kinase B
BF: bone fracture
BMD: bone mineral density
BSA: bovine serum albumin
BV/TV: bone volume/tissue volume
CCK-8: cell counting kit-8
CAM: chick chorioallantoic membranes
DAPI: 4 ′, 6-diamidino-2-phenylindole
DO: distraction osteogenesis
EPCs: endothelial progenitor cells
EDTA: ethylene diamine tetraacetic acid
FTO: fat mass and obesity-associated protein
FBS: fetal bovine serum
GAPDH: glyceraldehyde-3-phosphate dehydrogenase
H&E: hematoxylin-eosin
μ CT: micro-computed tomography
m^6^A: *N*6-methyladenosine
METTL3: methyltransferase-like 3
NC: negative control
OE: overexpression
OCN: osteocalcin
PBS: phosphate-buffered saline
PVDF: polyvinylidene fluoride
PFA: paraformaldehyde
qRT-PCR: quantitative real-time polymerase chain reaction
RIPA: radio immunoprecipitation assay
SDS: sodium dodecyl sulfate
Tb. Sp: trabecular separation
Tb. N: trabecular number
Tb. Th: trabecular thickness
VEGF: vascular endothelial growth factor
WB: Western blotting
WTAP: Wilms tumor 1 associated protein
YTH: YT521-B homology.
