# Supplementary figures and images for: The RNA Methyltransferase METTL3 Promotes Endothelial Progenitor Cell Angiogenesis in Mandibular Distraction Osteogenesis via the PI3K/AKT Pathway
Source: Front Cell Dev Biol. 2021 Nov 1;9:720925. doi: 10.3389/fcell.2021.720925 (PMC8591310; doi:10.3389/fcell.2021.720925)

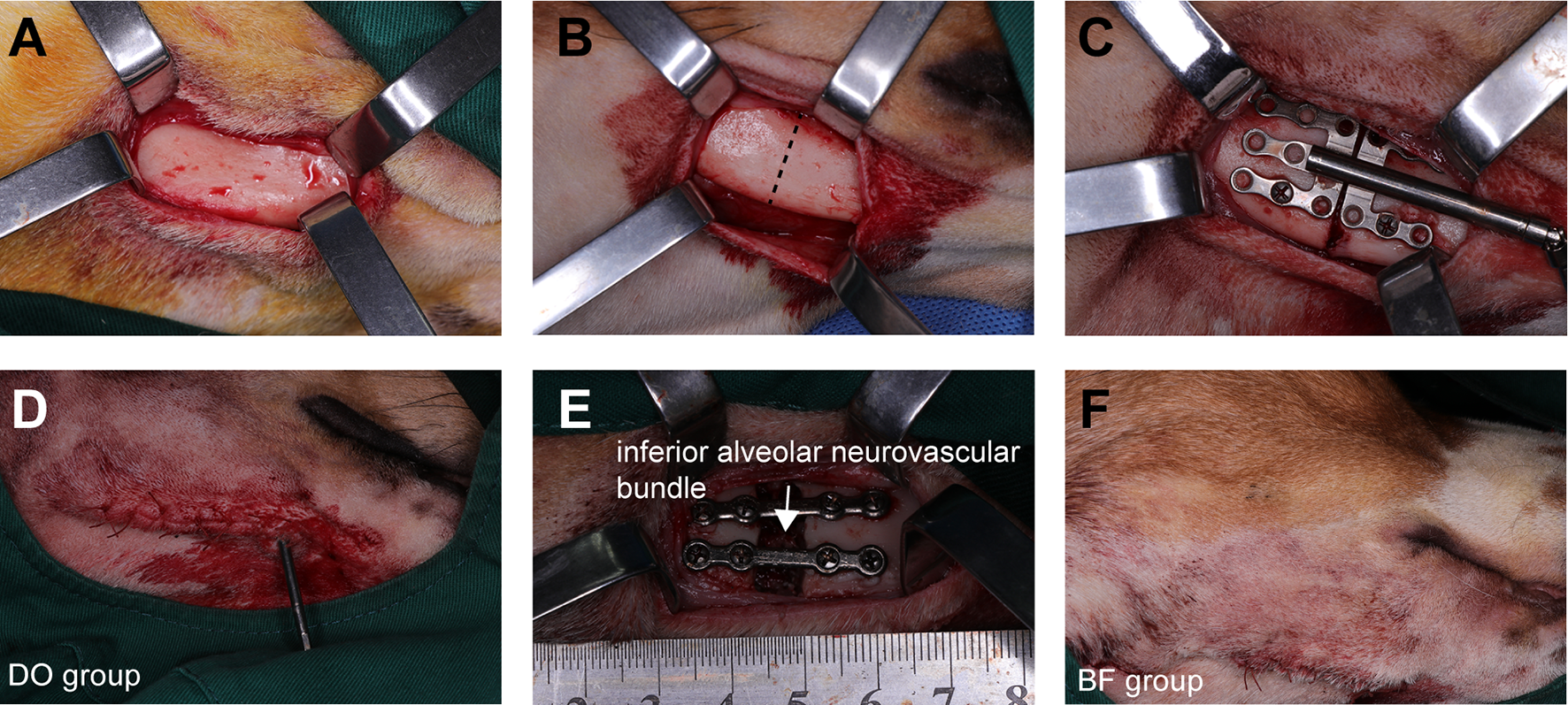

Supplement: Supplementary Figure 1 — Canine mandibular DO and BF model procedure. (A) Mandible exposure. (B) Osteotomy. (C) Distraction device installation. (D) Skin suturing (DO group). (E) Titanium plate and nail installation following osteotomy (BF group). (F) Mandibular skin closure. [file Image_1.TIF]

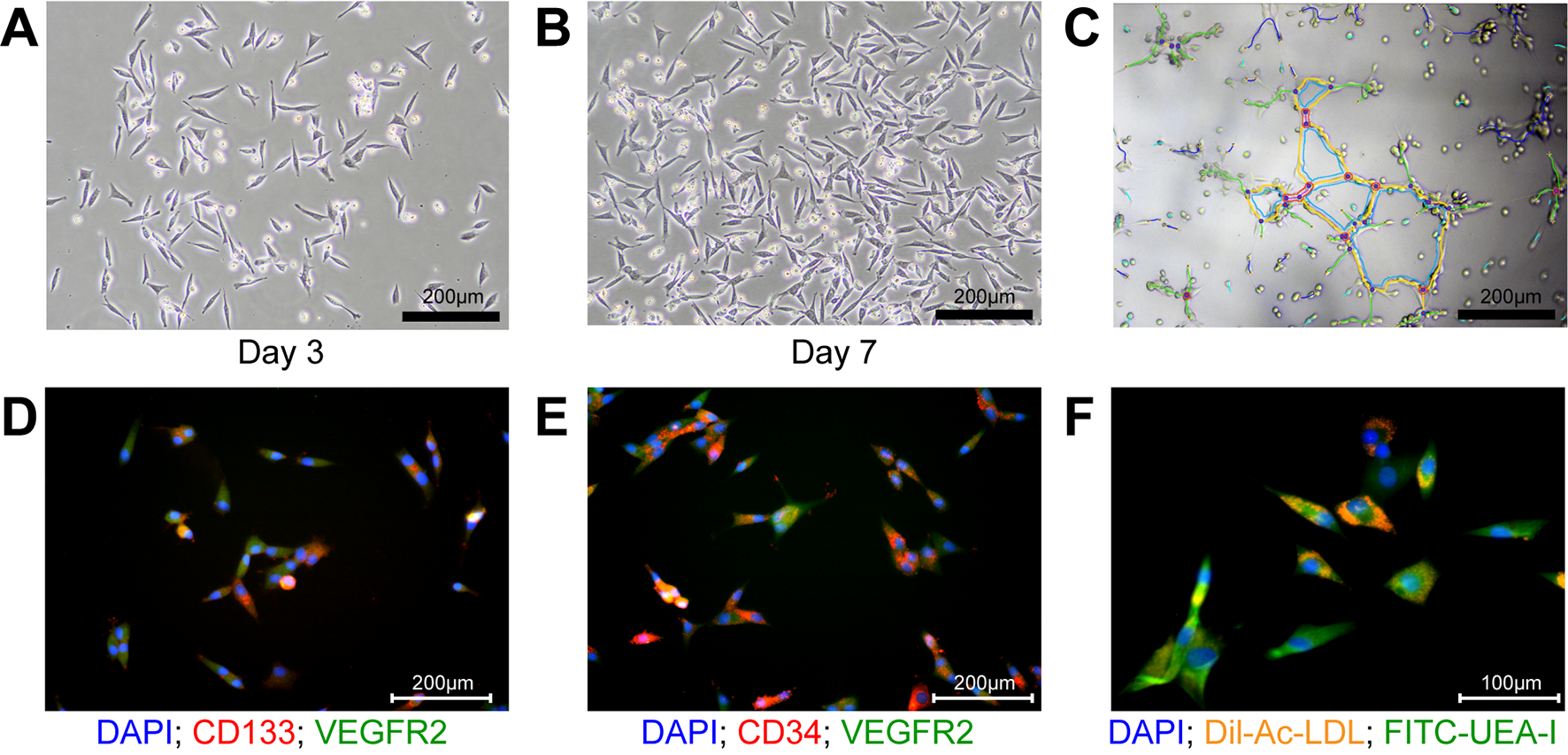

Supplement: Supplementary Figure 2 — Endothelial progenitor cells (EPC) Characterization. (A–C) Changes and tube formation for isolated EPCs were assessed over time. Following a 3-day culture period, a limited number of adherent cells were evident (A), with limited proliferation (B). The angiogenic activity of these EPCs was assessed via tube formation assay (C). (D–F) Canine EPCs were identified by staining for CD133 (Red), CD34 (Red), and VEGFR2 (Green) on the cell surface (D,E). Dil-ac-LDL uptake (Orange) and FITC-UEA-1 binding (Green) were assessed to gauge EPC functionality (F). [file Image_2.TIF]

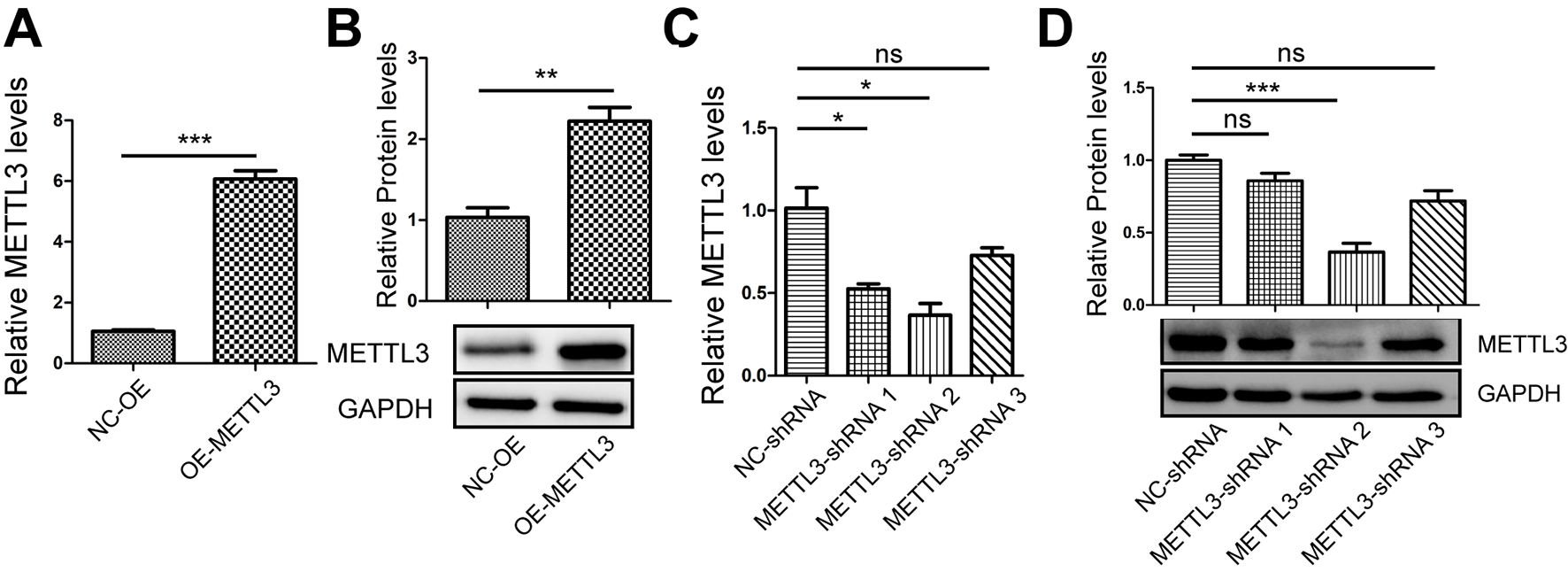

Supplement: Supplementary Figure 3 — Methyltransferase-like 3 (METTL3) knockdown or overexpression in EPCs. (A,B) METTL3 overexpression was confirmed by qRT-PCR (A) and Western blotting (B). (C,D) Relative EPC METTL3 mRNA (C) and protein (D) levels following NC-shRNA, METTL3-shRNA1, METTL3-shRNA2, or METTL3-shRNA3 transfection. Of these shRNAs, shRNA2 was the most efficacious and was therefore used for subsequent experiments. nsP > 0.05, ∗P < 0.05, ∗∗P < 0.01, ∗∗∗P < 0.001; n = 3 in each independent experiments. [file Image_3.TIF]
